# Comparable antibacterial effects and action mechanisms of silver and iron oxide nanoparticles on *Escherichia coli* and *Salmonella typhimurium*

**DOI:** 10.1038/s41598-020-70211-x

**Published:** 2020-08-04

**Authors:** Lilit Gabrielyan, Hamlet Badalyan, Vladimir Gevorgyan, Armen Trchounian

**Affiliations:** 1 Department of Medical Biochemistry and Biotechnology, Russian-Armenian University, 0051 Yerevan, Armenia; 2 Department of Biochemistry, Microbiology and Biotechnology, Biology Faculty, Yerevan State University, 0025 Yerevan, Armenia; 3 Department of General Physics and Astrophysics, Yerevan State University, 0025 Yerevan, Armenia; 4 Department of Technology for Materials and Electronic Technique Structures, Russian-Armenian University, 0051 Yerevan, Armenia

**Keywords:** Biochemistry, Biophysics, Microbiology

## Abstract

The current research reports the antibacterial effects of silver (Ag) and citric acid coated iron oxide (Fe_3_O_4_) NPs on *Escherichia coli* wild type and kanamycin-resistant strains, as well as on *Salmonella typhimurium* MDC1759. NPs demonstrated significant antibacterial activity against these bacteria, but antibacterial effect of Ag NPs is more pronounced at low concentrations. Ag NPs inhibited 60–90% of *S. typhimurium* and drug-resistant *E. coli.* The latter is more sensitive to Fe_3_O_4_ NPs than wild type strain: the number of bacterial colonies is decreased ~ 4-fold. To explain possible mechanisms of NPs action, H^+^-fluxes through the bacterial membrane and the H^+^-translocating F_O_F_1_-ATPase activity of bacterial membrane vesicles were studied. *N,N′-*Dicyclohexylcarbodiimide (DCCD)-sensitive ATPase activity was increased up to ~ 1.5-fold in the presence of Fe_3_O_4_ NPs. ATPase activity was not detected by Ag NPs even in the presence of DCCD, which confirms the bactericidal effect of these NPs. The H^+^-fluxes were changed by NPs and by addition of DCCD. H_2_ yield was inhibited by NPs; the inhibition by Ag NPs is stronger than by Fe_3_O_4_ NPs. NPs showed antibacterial effect in bacteria studied in concentration-dependent manner by changing in membrane permeability and membrane-bound enzyme activity. The F_O_F_1_-ATPase is suggested might be a target for NPs.

## Introduction

In recent years, the increase of the bacterial infections and the development of antimicrobial resistance have been avowed as one of the key problems of the biomedicine of twenty-first century, which require a finding of novel antibacterial agents. Nanoparticles (NPs) are considered a great alternative to antibiotics, since they show a high potential to solve the problem of antibiotic resistance. Various NPs are used in biomedicine, pharmaceutics, cosmetics, food industry, textile coating, etc.^1–5^.

In particular, silver (Ag) NPs and iron oxide (Fe_3_O_4_) have attracted much attention in biomedical field^4–9^. Ag NPs show a pronounced antibacterial activity not only against various pathogens, but also against bacterial biofilms^10–12^. Effect of NPs depends on the particle size, shape, charge, surface characteristics and magnetic properties^1,13,14^. For example, at the same concentration 10 nm Ag NPs are more effective than 100 nm Ag NPs against methicillin-resistant *Staphylococcus aureus*^15^. Moreover, Ag NPs demonstrate the stronger inhibitory effect than other NPs^11,16^. Ag NPs can physically interact with the cell surface of bacteria due to their large surface area, providing better interaction with bacteria^13^. Franchi et al.^10^ reported that Ag NPs can damage bacterial cell membranes, which provide to increasing of permeability of bacterial membrane. The Ag NPs can preferably affect the respiratory chain in bacterial cells^16^.

Iron oxide NPs such as Fe_3_O_4_ and γ-Fe_2_O_3_ NPs due to their super paramagnetic, high magnetic susceptibility and other properties are promising agents for biomedical applications. Nowadays, they have been used in drug delivery systems to deliver various compounds such as peptides, DNA molecules, chemotherapeutic, and hyper-thermic drugs^1,17,18^. These systems have a potential to minimize the side-effects and the required concentration of drugs, as well as decrease the damage of normal tissues. In these systems, magnetic targeting of drug delivery is considered as the most efficient way^17^.

The antimicrobial action of NPs is probably a result of their interaction with bacterial membrane, which can lead to alterations of membrane-bound mechanisms, membrane damage and the bacterial death^9,19–21^. However, the antibacterial mechanisms of NPs action are not fully explored and in this case, the study of the effects of NPs on various bacteria is important for finding out of the NPs' possible action mechanisms.

It is known, that susceptibility of microorganisms to NPs depends not only on NPs’ characteristics, but also on stabilizer type. Due to the tendency of NPs to aggregation, their use requires the stabilization. Stabilizer, which used for the magnetic NPs, can affect the properties of NPs^22–27^. Oleic acid was suggested as a promising stabilizer for magnetic NPs^22–24^. Oleic acid can form a protective monolayer around the NPs, which is necessary for monodispersed magnetic NPs formation^22–24^. However, stabilization of magnetic NPs with oleic acid makes the NPs soluble only in organic solvents and therefore limits their use in biomedicine^22,23^. By the way, for biomedical applications in aqueous media, the hydrophobic stabilizer needs to be replaced by a hydrophilic one^23^.

In our previous studies oleic acid coated Fe_3_O_4_ NPs’ effects on Gram-negative *Escherichia coli* BW25113, ampicillin-resistant *E. coli* DH5α-pUC18, kanamycin-resistant *E. coli* pARG-25 stains and Gram-positive *Enterococcus hirae* ATCC9790 growth and membrane-associated mechanisms have been investigated^20,21^. Our results showed that the Fe_3_O_4_ NPs demonstrate different effects on Gram-negative and Gram-positive bacteria. Gram-positive *E. hirae* displayed higher susceptibility to NPs than Gram-negative *E. coli*, because the components of cell wall of Gram-positive and Gram-negative bacteria have different pathways for NPs adsorption^2,7^. Stabilizer oleic acid had no any effect on growth properties of investigated bacteria.

Citric acid is another stabilizer, which able to stabilize magnetic NPs, but there are few data in literature about citric acid coated NPs antibacterial properties. It has been shown that treating NPs by citric acid in aqueous solution stabilized the NPs surfaces^25–27^. The citric acid coated NPs remained stable in aqueous solution even in a week, and this stability is sustained under an electric field in aqueous media^27^. Study of citric acid stabilized NPs antibacterial properties is very important for the regulation of various bacteria growth in biomedicine and biotechnology.

In the present work, the antibacterial effects and possible mechanisms of Ag NPs and citric acid coated Fe_3_O_4_ NPs on *E. coli* wild type and drug-resistant strains as well as in comparison with *Salmonella typhimurium* MDC1759, have been studied for the first time. Additional characteristics of NPs were determined. In order to analyze the role of different membrane-bound systems and to find out probable targets H^+^-translocating F_O_F_1_-ATPase activity, H^+^-fluxes through the bacterial membrane and H_2_ yield were also investigated.

## Results

### Growth characteristics of ***E. coli*** wild type and drug-resistant strains, and ***S. typhimurium*** in the presence of Ag NPs and citric acid coated Fe_3_O_4_ NPs

*Escherichia* and *Salmonella* species are the most common foodborne human pathogens causing to various types of illnesses^9,28–30^. In this case, it is important to study the effect of Ag and Fe_3_O_4_ NPs (coated by citric acid) on *E. coli* wild type and antibiotic-resistant strains, as well as *S. typhimurium* MDC1759 strain, for revealing the action mechanisms. The growth parameters of *E. coli* wild type K-12, kanamycin-resistant pARG-25, and *S. typhimurium* MDC1759 strains in the presence of Ag and Fe_3_O_4_ NPs have been investigated. Bacteria grown in the presence of kanamycin (50 μg mL^−1^) were used as positive control. The negative controls are the strains, cultivated without antibiotic.

**Figure 1:**
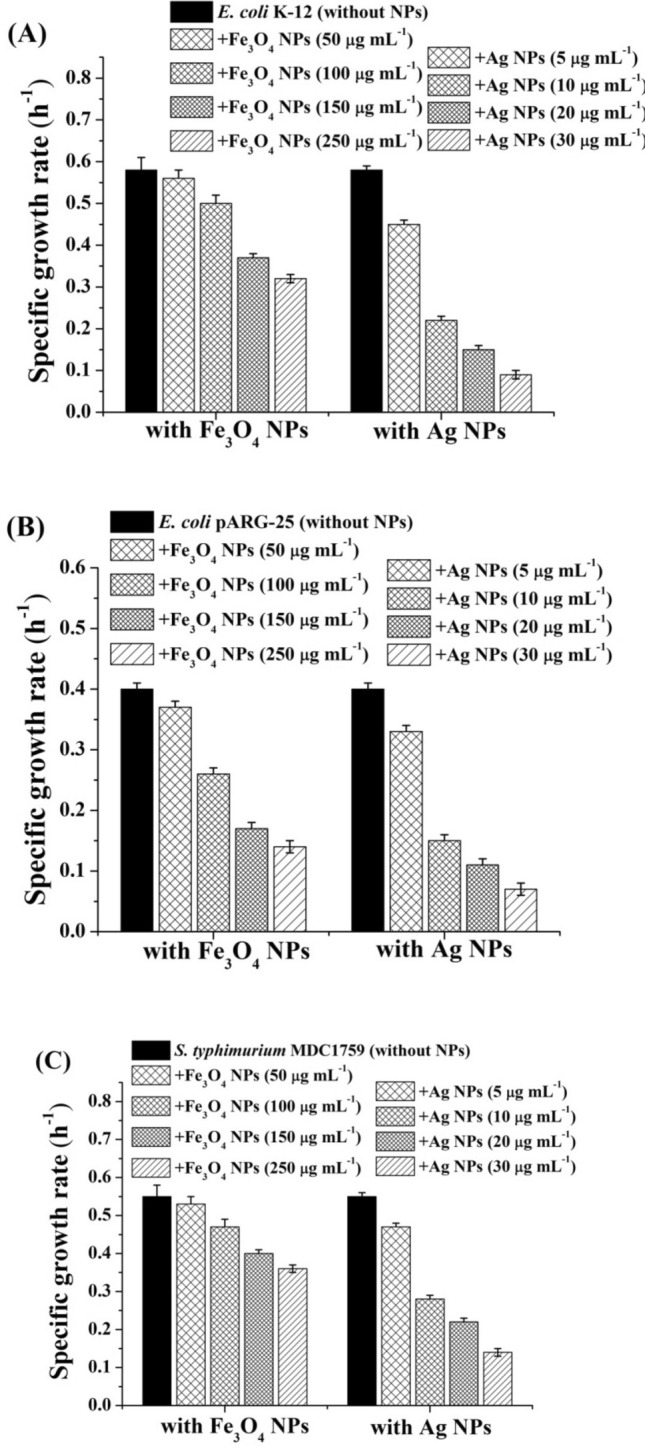
Specific growth rates of *E. coli* K-12 (**A**) kanamycin-resistant *E. coli* pARG-25 (**B**), and *S. typhimurium* MDC1759 (**C**) strains in the presence of citric acid coated Fe_3_O_4_ and Ag NPs various concentrations. Control was without nanoparticles addition.

The results obtained show antibacterial effects of Ag and Fe_3_O_4_ NPs. The intensity of NPs effects depends on the type of NPs, their concentrations and bacterial strains. Both NPs showed inhibitory effect on *E. coli* growth (Fig. 1). Moreover, the effect of Fe_3_O_4_ NPs also depends on the type of stabilizer. Stabilization of NPs by various compounds enhances an antimicrobial activity of NPs^8,26,31^.

In the presence of citric acid coated Fe_3_O_4_ NPs (100–250 μg mL^−1^) inhibition of *E. coli* K-12 growth was observed (Fig. 1A). NPs demonstrated concentration dependent effect on *E. coli* growth (Fig. 3). The specific growth rate of *E. coli* K-12 at NPs' concentration 50 μg mL^−1^ was similar to the control, and decreased ~ 1.2-fold at 100 μg mL^−1^ NPs (Fig. 3). The maximal inhibitory effect has been obtained at the concentration 250 μg mL^−1^, which led to the decrease in bacterial specific growth rate by ~ 2.0-fold, indicating the bacteriostatic effect of the Fe_3_O_4_ NPs. The antibacterial effect of Fe_3_O_4_ NPs may be due to several mechanisms. ROS together with superoxide radicals (O^2−^), hydroxide radical (OH^−^) and singlet oxygen formed by Fe_3_O_4_ NPs could be the reason of inhibition. Similar results were obtained in other studies showing the antibacterial activity of Fe_3_O_4_ NPs against *E. coli*^19^. In our previous study oleic acid coated Fe_3_O_4_ NPs show antibacterial activity against *E. coli* BW25113 strain^21^. However, the citric acid coated Fe_3_O_4_ NPs demonstrate more pronounced antibacterial activity than Fe_3_O_4_ NPs coated by oleic acid at the same concentration. The maximal inhibitory effect of oleic acid coated Fe_3_O_4_ NPs has been observed at the 500 μg mL^−1^ concentration^21^. Moreover, citric acid does not show any effect on growth rates of investigated bacteria (not shown).

Ag NPs show more pronounced bactericidal effect than Fe_3_O_4_ NPs (Fig. 1). Moreover, Ag NPs display more expressed antibacterial effect at low concentrations. In the presence of 10 μg mL^−1^ Ag NPs a 2.6-fold suppression of growth of *E. coli* K-12 was observed (see Fig. 1A). The 20–30 μg mL^−1^ Ag NPs led to 4.0–6.5-fold decrease in bacterial growth rate, indicating the bactericidal effect of these concentrations.

Moreover, kanamycin-resistant *E. coli* pARG-25 strain had more susceptibility to Fe_3_O_4_ NPs than *E. coli* wild type strain (Fig. 1B). In the presence of 100–250 μg mL^−1^ Fe_3_O_4_ NPs a 1.5–3-fold inhibition of bacterial growth was observed (see Fig. 1B). Similar results were obtained with ampicillin-resistant *E. coli* DH5α-pUC18 strain (not shown). Citric acid coated Fe_3_O_4_ NPs demonstrated more noticeable antibacterial effect than Fe_3_O_4_ NPs stabilized by oleic acid^20^. In the case of Ag NPs, as seen in Fig. 1B, there were no obvious differences between antibacterial effect of these NPs on wild type and drug-resistant bacteria.

The effect of Fe_3_O_4_ NPs on *S. typhimurium* MDC1759 growth rate is the same with the action of iron oxide NPs on *E. coli* K-12 strain (Fig. 1C). Antibacterial effect of Ag NPs on *S. typhimurium* MDC1759 was less pronounced than on *E. coli* both strains (Fig. 1C). At the same time, *S. typhimurium* demonstrated susceptibility to the high concentrations of Ag NPs: 20 μg mL^−1^ Ag NPs led to ~ 2.5-fold decrease in growth specific rate (Fig. 1C).

**Figure 2:**
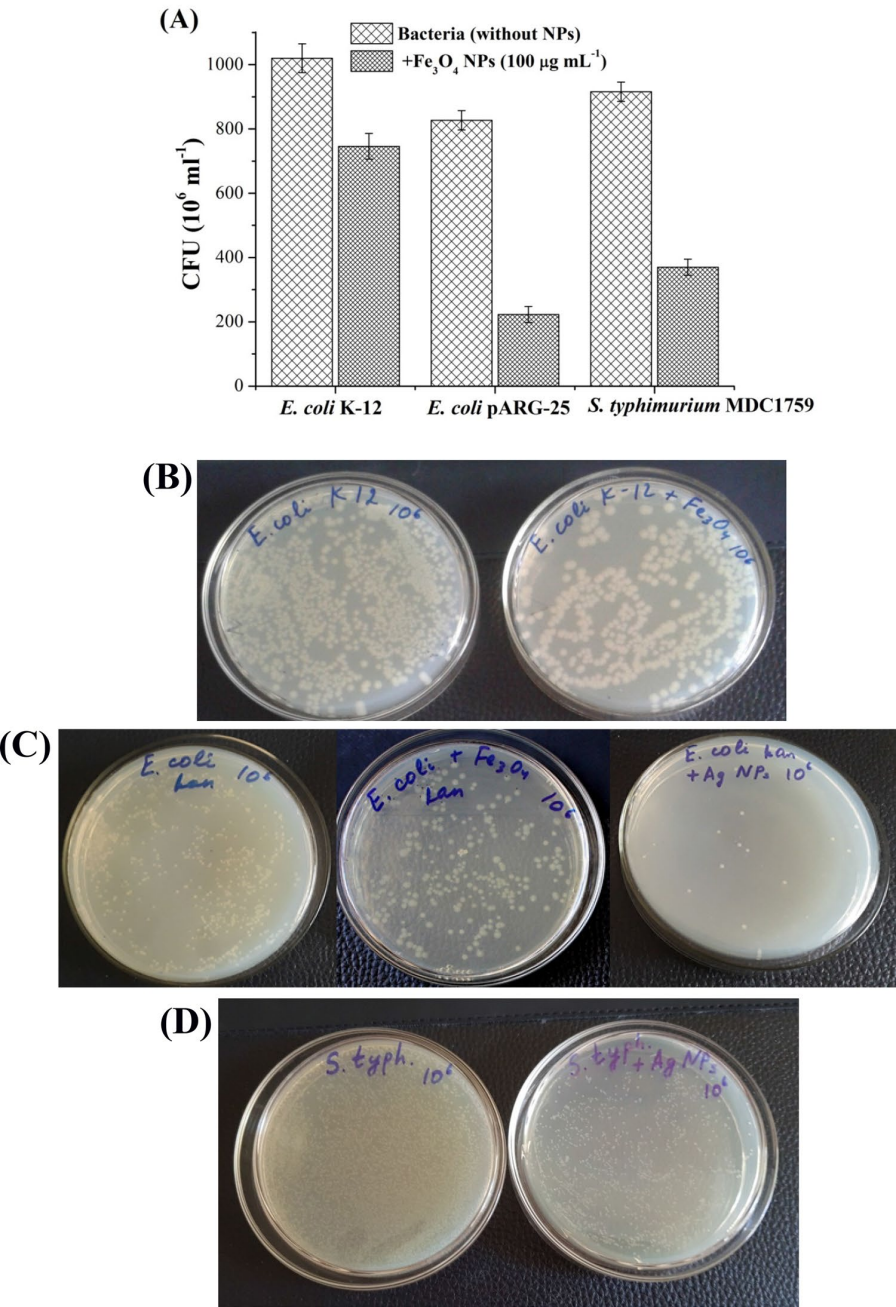
The number of viable colonies of *E. coli* K-12, kanamycin-resistant *E. coli* pARG-25, and *S. typhimurium* MDC1759 strains grown in the presence of citric acid coated Fe_3_O_4_ NPs (100 μg mL^−1^) (**A**). The colonies of *E. coli* K-12 grown in the absence and presence of Fe_3_O_4_ NPs (100 μg mL^−1^) (**B**). The colonies of *E. coli* pARG-25 grown in the absence and presence of Fe_3_O_4_ NPs (100 μg mL^−1^) and Ag NPs (10 μg mL^−1^) (**C**). The colonies of *S. typhimurium* MDC1759 cultivated in the absence and presence of Ag NPs (10 μg mL^−1^) (**D**).

**Figure 3:**
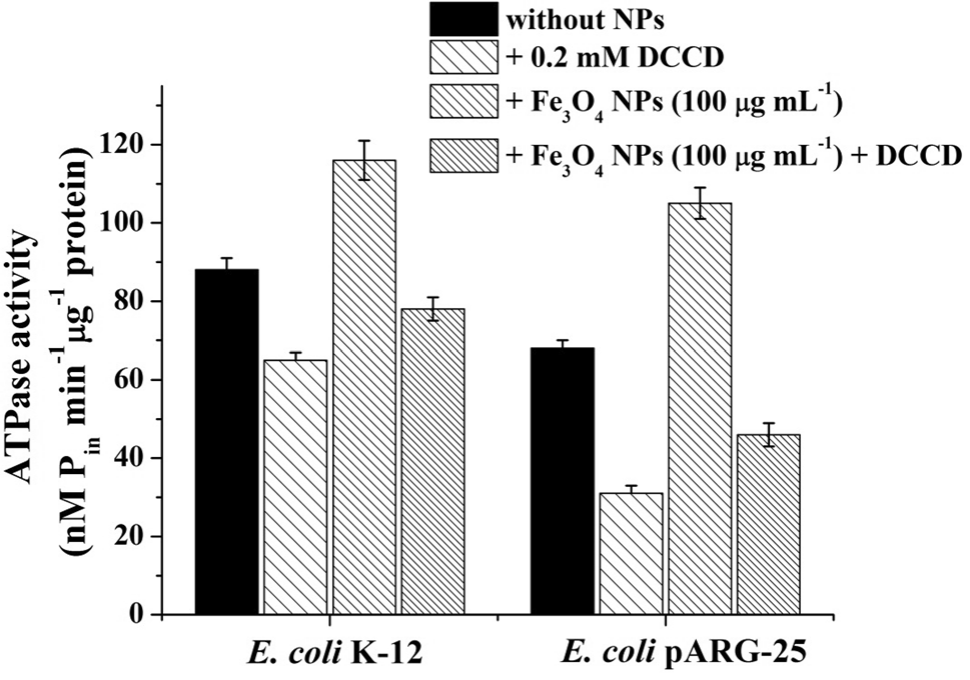
ATPase activity of *E. coli* K-12 and kanamycin-resistant *E. coli* pARG-25 membrane vesicles in the presence of citric acid coated Fe_3_O_4_ NPs (100 μg mL^−1^) and DCCD (0.2 mM). Control was without NPs addition.

Figures 2 and 3 display the number of viable colonies of *E. coli* K-12 and pARG-25, and *S. typhimurium* MDC1759, grown in the absence and presence of 100 μg mL^−1^ Fe_3_O_4_ NPs. CFU of *E. coli* K-12 was decreased 1.3-fold in the presence of Fe_3_O_4_ NPs (Fig. 2A,B). At the same time, *S. typhimurium* MDC1759 and drug-resistant *E. coli* strain has more susceptibility to Fe_3_O_4_ NPs (stabilized by citric acid) than wild type strain: CFU was decreased 2.5–4-fold, respectively (see Fig. 2A). In the case of kanamycin-resistant *E. coli* strains Fe_3_O_4_ NPs coated by citric acid showed noticeable antibacterial effect in comparison with oleic acid stabilized Fe_3_O_4_ NPs^20^. Ag NPs inhibited ~ 60 to 90% of *S. typhimurium* MDC1759 and drug-resistant *E. coli* viable colonies (Fig. 2C,D).

Thus, NPs show significant antibacterial activity against used bacteria. Different effects of Fe_3_O_4_ and Ag NPs on bacterial growth may be due to the difference in the interaction between the bacterial cells and NPs^2,11^. Small size of NPs can contribute to their antibacterial activity^7,11^. NPs can interact closely with bacterial membranes and the inactivation of bacteria could be due to their penetration into the bacterial cell^1,9,11^.

### H^+^-fluxes through the bacterial membrane and H^+^-translocating ATPase activity in ***E. coli*** wild type and drug-resistant strains in the presence of Ag NPs and citric acid coated Fe_3_O_4_ NPs

In order to explain the possible mechanisms and find out probable targets of the effect of NPs changes in H^+^-fluxes through the bacterial membrane and H^+^-translocating F_O_F_1_-ATPase activity in *E. coli* wild type and drug-resistant strains were investigated.

H^+^-coupled membrane transport has been determined in *E. coli* wild type, drug-resistant strains, and *S. typhimurium* in the absence and presence of citric acid stabilized Fe_3_O_4_ and Ag NPs (Table 1). Fe_3_O_4_ NPs suppressed energy-dependent H^+^-efflux in *E. coli* K-12 and pARG-25 strains ~ 3.0 to and ~ 1.3-folds, respectively (see Table 1). H^+^-fluxes also decreased in the presence of DCCD, an inhibitor of the H^+^-translocating ATPases (see Table 1). The presence of Ag NPs in the assay medium led to the increase in H^+^ fluxes, in comparison with Fe_3_O_4_ NPs. A more noticeable effect was observed in *E. coli* K-12: H^+^-fluxes were increased ~ 1.2-fold. The effect of Ag NPs on *E. coli* pARG-25 was weaker in comparison with *E. coli* wild type strain (see Table 1). At the same time, in the presence of Ag NPs the H^+^-fluxes were increased even in the presence of DCCD, indicating that Ag NPs affected the bacterial membrane leading to changes in membrane structure and permeability. The same results were obtained with *E. coli* BW 25113^1^. In case of *S. typhimurium* both NPs suppressed energy-dependent H^+^-efflux even by addition of DCCD (Table 1).

**Table 1:** The changes of H^+^-fluxes across the bacterial membranes of *E. coli* K-12, drug-resistant *E. coli* pARG-25 and *S. typhimurium* MDC1759 strains in the presence of citric acid coated Fe_3_O_4_ (100 μg mL^−1^) stabilized by citric acid, and Ag NPs (10 μg mL^−1^).

Bacteria and conditions*	H^+^-fluxes (mmol H^+^ min^−1^ (10^10^ cells)^−1^	H^+^-fluxes + DCCD** (mmol H^+^ min^−1^ (10^10^ cells)^−1^
*E. coli* K-12 (without NPs)	2.50 ± 0.02	1.10 ± 0.02
*E. coli* K-12 + Fe_3_O_4_ NPs	0.75 ± 0.01 P*** < 0.01	0.68 ± 0.01 *P* < 0.01
*E. coli* K-12 + Ag NPs	2.87 ± 0.02 *P* < 0.05	1.25 ± 0.02 *P* < 0.05
*E. coli* pARG-25 (without NPs)	2.32 ± 0.02	1.05 ± 0.02
*E. coli* pARG-25 + Fe_3_O_4_ NPs	1.80 ± 0.02 *P* < 0.01	0.98 ± 0.01 *P* < 0.01
*E. coli* pARG-25 + Ag NPs	2.55 ± 0.02 *P* < 0.05	1.15 ± 0.01 *P* < 0.01
*S. typhimurium* MDC1759 (without NPs)	1.50 ± 0.02	0.83 ± 0.01
*S. typhimurium* MDC1759 + Fe_3_O_4_ NPs	1.16 ± 0.02 *P* < 0.01	0.27 ± 0.02 *P* < 0.01
*S. typhimurium* MDC1759 + Ag NPs	1.32 ± 0.02 *P* < 0.01	0.34 ± 0.01 *P* < 0.01

*The bacteria were washed and transferred into Tris–phosphate buffer; bacterial cells were treated with NPs for 10 min.

**The bacterial cells were treated with 0.2 mM DCCD for 10 min.

***P is difference between the values of experimental simples and appropriate control.

The membrane-bound H^+^-translocating F_O_F_1_-ATPase activity of *E. coli* membrane vesicles was analyzed in the presence of citric acid coated Fe_3_O_4_ and Ag NPs to reveal their effects on the ATPase. In facultative anaerobic bacteria, such as *E. coli*, the H^+^-translocating ATPase is reversible depending on bacterial growth conditions^32^. During the preparation of membrane vesicles, spheroplasts from Gram-negative *E. coli* were obtained.

Fe_3_O_4_ NPs led to a ~ 1.3 to 1.5-fold increase in total F_O_F_1_-ATPase activity in membrane vesicles of *E. coli* K-12 and pARG-25, respectively (Fig. 3). DCCD-sensitive ATPase activity was increased ~ 1.2- and 1.5-folds in *E. coli* K-12 and pARG-25, respectively (see Fig. 3). In the case of Ag NPs, ATPase activity was not detected even in the presence of DCCD (no shown), which confirms the bactericidal effect of these NPs. Therefore, the effect of NPs on the ATPase can be responsible for the antibacterial effect.

NPs can directly affect the F_O_F_1_-ATPase, because ATPase activity was changed even in the absence of DCCD, an inhibitor of H^+^-translocating systems; or this effect can be intermediated by membrane-associated formate hydrogen lyase (FHL) complexes, which are responsible for H_2_ production in *E. coli*. The bacterial membrane permeability can be changed during the bacterial growth in the presence of NPs.

### The redox potential changes and H_2_ production ability in ***E. coli*** wild type and drug-resistant strains in the presence of Ag NPs and citric acid coated Fe_3_O_4_ NPs

**Figure 4:**
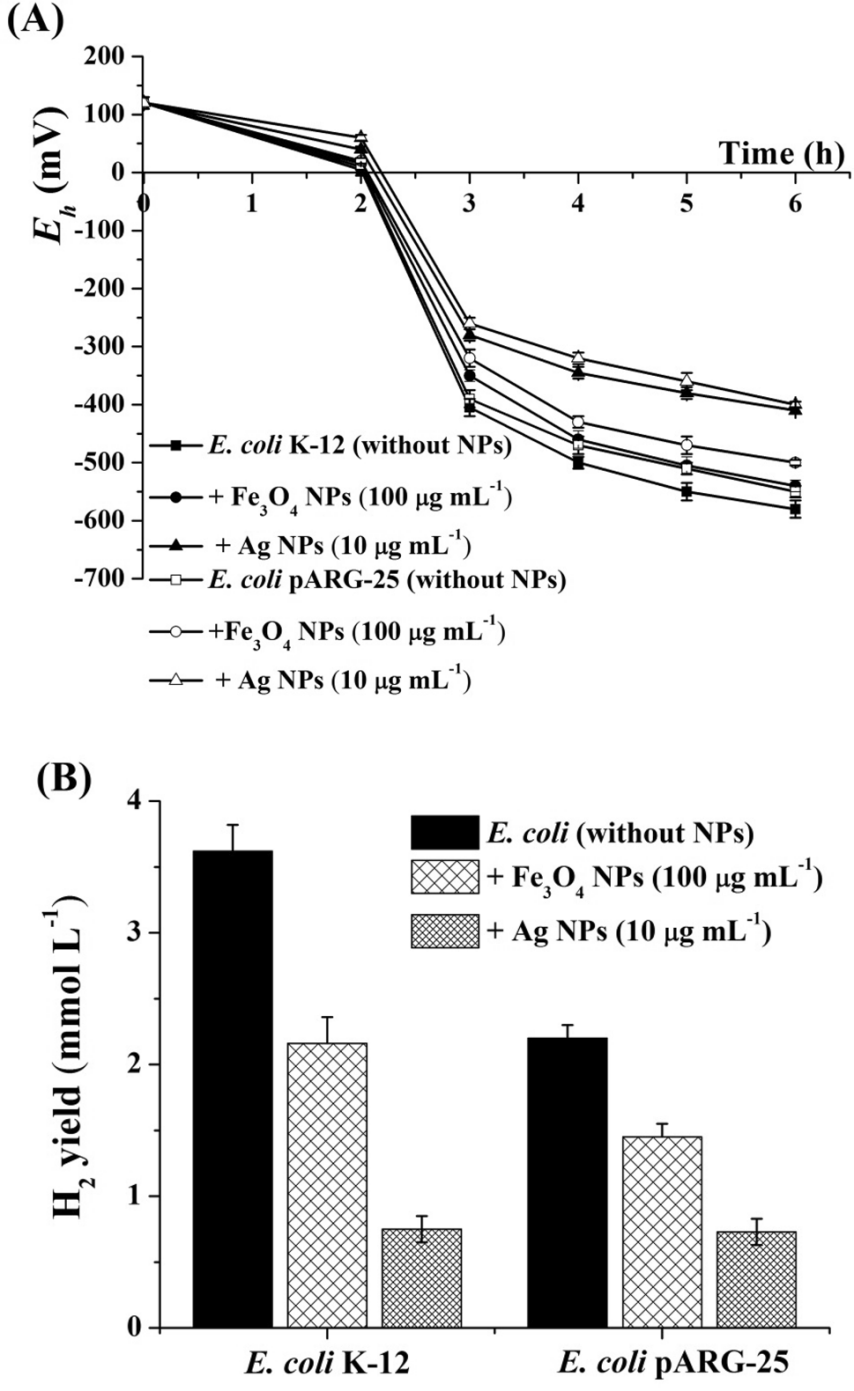
The changes of redox potential (**A**) and the H_2_ yield (**B**) in *E. coli* K-12 and kanamycin-resistant *E. coli* pARG-25 strains during anaerobic growth in the presence of citric acid coated Fe_3_O_4_ (100 μg mL^−1^) and Ag NPs (10 μg mL^−1^). Control was without NPs addition.

The redox potential (*E*_*h*_) is an important factor, which characterizes the metabolic activity of bacteria under various growth conditions^1^. To reveal the action mechanisms of Ag and Fe_3_O_4_ NPs on both *E. coli* strains, the kinetics of *E*_*h*_ during the bacterial growth has been studied. The anaerobic growth (6 h) of bacteria was accompanied by a drop in the value of *E*_*h*_ from a positive value (+ 120 ± 10 mV) at the beginning of the growth lag phase to a negative value (− 580 ± 15 mV) for *E. coli* K-12 and (− 550 ± 10 mV) for *E. coli* pARG-25 (Fig. 4A). This decrease indicates an enhancement of reduction processes that characterizes bacterial metabolism under anaerobic conditions and the generation of H_2_^1,20^. The addition of NPs resulted in a delayed redox potential drop (Fig. 4A). In the presence of Fe_3_O_4_ NPs value of the *E*_*h*_ decreased up to (− 540 ± 10 mV) for *E. coli* K-12 and (− 500 ± 10 mV) for *E. coli* pARG-25 (see Fig. 4A). Ag NPs exhibited more pronounced effect: in the presence of 10 μg mL^−1^ Ag NPs, the *E*_*h*_ values ​​of *E. coli* K-12 and pARG-25 cells were decreased up to (− 410 ± 5 mV) and (− 400 ± 5 mV), respectively (see Fig. 4A). The inhibition of bacterial growth in the presence of NPs can be coupled with *E*_*h*_ or with a direct effect of NPs on the bacterial membrane.

A correlation between the decrease of *E*_*h*_ and production of H_2_ is shown for *E. coli,* which can generate H_2_ under the action of FHL complexes^32,33^. During glucose fermentation, H_2_ production was determined during 6 h anaerobic growth in both strains: 3.62 and 2.20 mmol H_2_ L^−1^ in *E. coli* K-12 and pARG-25 cells, respectively (Fig. 4B). The H_2_ production by *E. coli* coupled with the activity of membrane-associated H_2_-producing enzymes—hydrogenases, which are involved in H_2_ metabolism in *E. coli*^21,32,33^. Ag and Fe_3_O_4_ NPs inhibited H_2_ yield in both *E. coli* strains (see Fig. 4B). H_2_ production by both strains in the presence of 100 μg mL^−1^ citric acid coated Fe_3_O_4_ NPs was ~ 1.5 to 1.7-fold lower in comparison with control cells (see Fig. 4B). Citric acid coated Fe_3_O_4_ NPs demonstrated more noticeable effect on H_2_ yield than NPs stabilized by oleic acid^20,21^. In the presence of 100 μg mL^−1^ oleic acid coated NPs H_2_ yield in *E. coli* pARG-25 was decreased 1.2-fold in comparison with control, and was not changed significantly in *E. coli* BW25113^20,21^. In the presence of Ag NPs H_2_ yield in *E. coli* K-12 was ~ 5.0-fold lower than H_2_ yield in control cells (see Fig. 4B).

The results point out that NPs show antibacterial effect in both bacteria in concentration-dependent manner by changing membrane-bound enzyme activity.

## Discussion

Nowadays, due to the development of drug-resistant bacteria, the search of new more effective antibacterial agents required. Various NPs are suggested as novel antimicrobial agents against different pathogens owing to their unique physicochemical properties^1,4–7^. As it was shown in our previous work, oleic acid coated Fe_3_O_4_ NPs display different effect on the growth properties and membrane activity of Gram-negative *Escherichia coli* BW 25113 and Gram-positive *Enterococcus hirae* ATCC 9790^21^. Iron oxide NPs demonstrate better antibacterial effect on Gram-negative, than on Gram-positive bacteria^21^. This effect can be coupled with the components of cell wall of Gram-positive and Gram-negative bacteria. Gram-positive bacteria have a thick peptidoglycan layer, teichoic acids and pores, which allow penetration of external molecules, including NPs^2^. In contrast, the cell wall of Gram-negative bacteria has a thin peptidoglycan layer between the cytoplasmic membrane and the outer membrane, which forms a penetration barrier for external molecules.

Stabilizer such as oleic acid can be used not only to prevent aggregation of magnetic NPs, but also for protection of NPs^22–24^. Citric acid also can be used to stabilize the magnetic NPs for biomedical application^25–27^.

In this study, antimicrobial effects and possible mechanisms of Fe_3_O_4_ NPs (coated by citric acid) and Ag NPs on *E. coli* K-12 wild type and pARG-25 kanamycin-resistant strains have been investigated. Citric acid coated Fe_3_O_4_ NPs exhibit significant antibacterial activity against *E. coli* both strains, but kanamycin-resistant *E. coli* pARG-25 strain is more susceptible to Fe_3_O_4_ NPs than wild type strain. Moreover, the effect of Fe_3_O_4_ NPs depended of stabilizer type. Citric acid coated Fe_3_O_4_ NPs demonstrated more noticeable action against drug-resistant bacteria than oleic acid stabilized NPs^20^. In the presence of 250 μg mL^−1^ citric acid coated Fe_3_O_4_ NPs a ~ threefold inhibition of bacterial growth was observed, whereas the same concentration of oleic acid coated NPs suppressed growth rate ~ 2-fold^20^.

Ag NPs exhibit a more pronounced bactericidal effect in comparison with Fe_3_O_4_ NPs. Moreover, Ag NPs have a more expressed antibacterial effect at low concentrations. The low concentration effect of Ag NPs has been reported by various researchers^10–12,34^. There were no obvious differences in bactericidal activity between *E. coli* wild type and drug-resistant strains, confirming that Ag NPs have a broad spectrum of action. Ag NPs are assumed to affect not only the growth of both *E. coli* strains, but also the energy-dependent H^+^-coupled membrane transport and ATPase activity in bacteria. The increase of H^+^-fluxes in the presence of DCCD indicated the significant effect of Ag NPs on the bacterial membrane structure and permeability. Probably Ag NPs change the permeability of the bacterial membrane and inhibit the cell respiration by penetrating via cell wall^9,10,14^. These studies confirm that the F_O_F_1_-ATPase, which plays a crucial role in bacterial metabolism, can be a sensitive target for metals NPs action. The effect of NPs used on the F_O_F_1_-ATPase can be responsible for the antibacterial effect, and this ATPase can be a target for NPs. Similar data were obtained in mammalian cells, where Ag NPs inhibited mitochondrial ATPase activity of rat liver cells^35^.

Different effects of Fe_3_O_4_ and Ag NPs on *E. coli* ATPase activity may be coupled to the differences in the interaction of NPs with bacterial cell wall. The antimicrobial activity of metal NPs is a result of NPs interaction with bacterial membranes and their penetration into the bacterial cell, causing membrane damage and bacterial death^2,7–9^.

Thus, metal NPs studied exhibited antibacterial activity against bacteria, including drug-resistant strains, and they can be applied in biomedicine for the treatment of various infections and in biotechnology and the food industry for controlling bacterial growth.

## Materials and methods

### Bacterial strains, cultivation conditions and growth determination

This study was performed with *S. typhimurium* MDC1759, *E. coli* K-12 wild type and kanamycin-resistant pARG-25 strains (Microbial Depository Center, National Academy of Science, Yerevan, Armenia). Bacteria grown in the presence of kanamycin (50 μg mL^−1^) were used as positive control. The negative controls are the strains, cultivated without antibiotic. These strains were cultivated in peptone medium at 37 °C and pH 7.5^20,21^. Anaerobic conditions, favorable for intestine microorganisms, including pathogenic ones, were maintained^20^. For creation of anaerobic conditions O_2_ was bubbled out from media by autoclaving at 120 °C for 20 min, and then bottles were closed by press caps. To reach anaerobic conditions the bottles were kept sealed to maintain anoxic conditions and all experiments were performed under strict absence of O_2_^20,33^.

The growth of bacteria was determined by measuring the optical density at 600 nm using Spectro UV–Vis Auto spectrophotometer (Labomed, Los Angeles, USA). The concentration of initial inoculum was 10^8^ of number of colony forming units (CFU) mL^−1^. Specific growth rate was determined as the quotient of ln2 division on doubling time of absorbance over the interval, when the logarithm of absorbance of the culture at 600 nm increased with time linearly (logarithmic growth phase), and it was expressed as h^−1^ as decribed^20,21^. Colloidal Ag NPs ("Silverton", "Tonus-Less", Armenia) in the concentration range from 5 to 30 μg mL^−1^ and Fe_3_O_4_ NPs (stabilized by citric acid) from 50 to 250 μg mL^−1^ were added to growth medium with inoculum.

### Characterization of Ag and Fe_3_O_4_ nanoparticles

**Figure 5:**
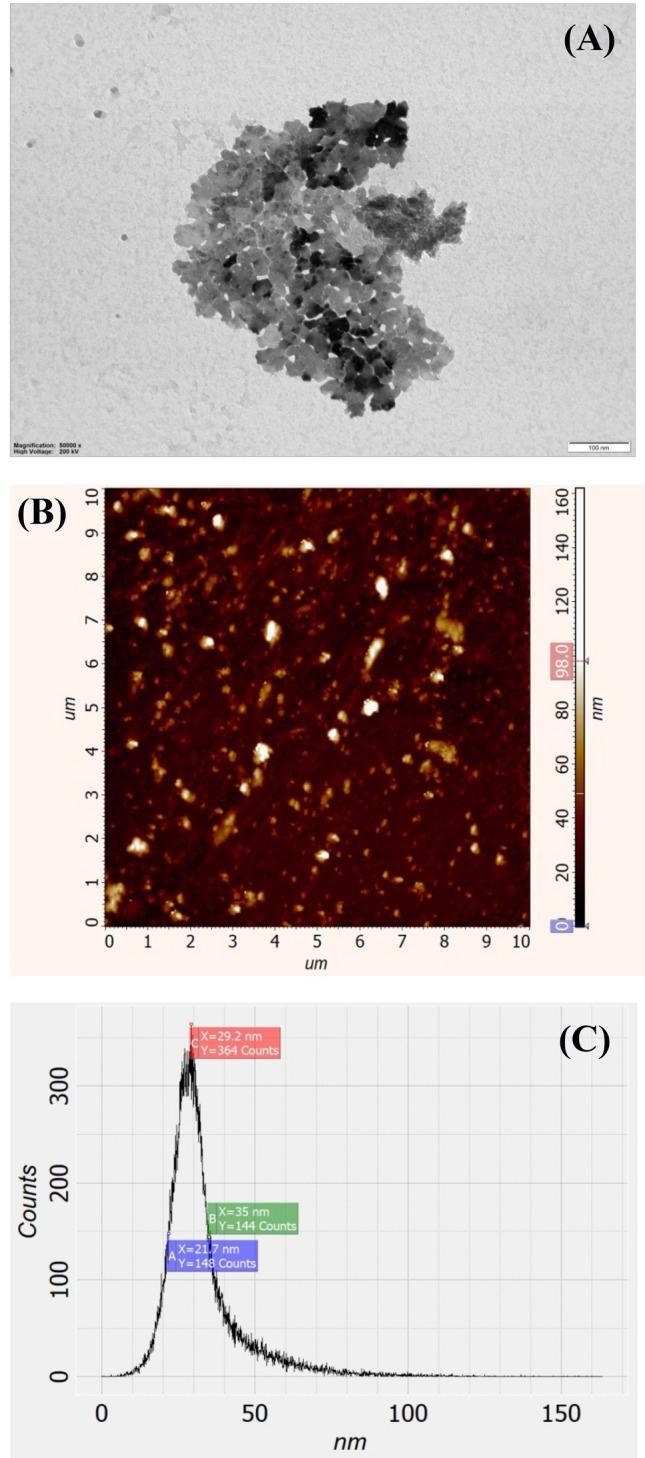
TEM image (**A**) and atomic force microscopy of Ag NPs using appropriate microscope (**B**). Ag NPs distribution in the colloid solution depending of their size (**C**). For details, see “Materials and methods”.

The structure, form and size of Ag NPs (synthesized by electrochemical method^36^) were investigated using TEM image and atomic force microscopy method by appropriate microscope (NT-MDT Nanoeducator 2, Russia). The data showed that Ag NPs have spherical form with average size of ~ 30 nm (Fig. 5).

**Figure 6:**
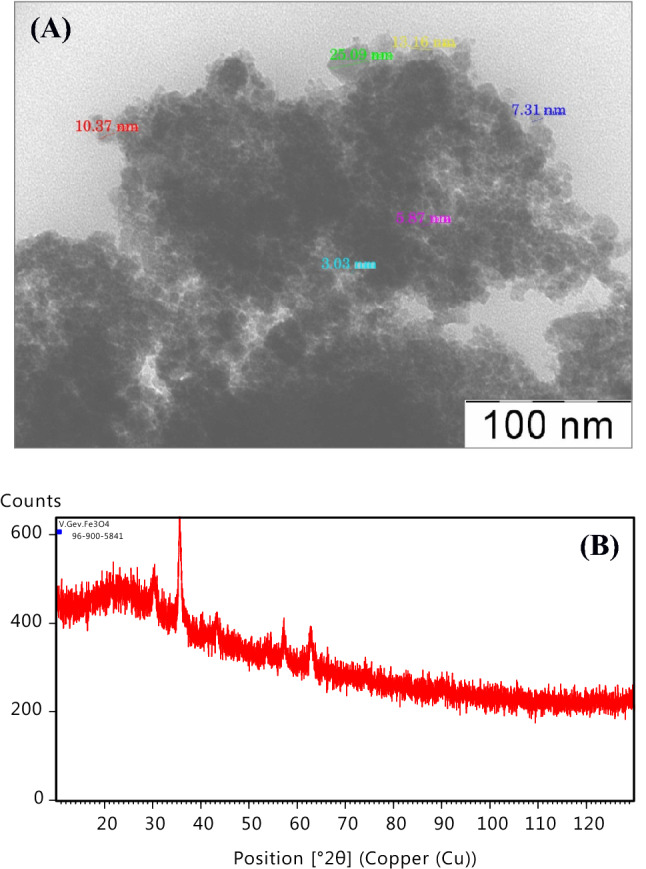
TEM image of synthesized Fe_3_O_4_ NP (**A**). The sizes of some nanoparticles indicated immediately in the figure. XRD pattern of the Fe_3_O_4_ NPs (**B**).

Fe_3_O_4_ NPs coated with citric were synthesized by co-precipitation method^20,37^. Fe_3_O_4_ NPs have round form and average size of 10 nm (Fig. 6). The synthesized Fe_3_O_4_ NPs were characterized also by X-ray powder diffraction (XRD) to determine the sample phases and average particle size of the dried powder. The XRD pattern of the sample was recorded on diffractometer system EMPYREAN using CuKα (λ = 1.5406 Å) radiation at room temperature in the range of 10° to 120° in the 2θ scale, Generator Settings 40 mA, 45 kV. The XRD patterns of the dried sample of Fe_3_O_4_ NPs and diffraction peaks parameters are shown in Fig. 6 and Table 2. XRD data can be used to distinguish the crystallinity and the average size of nanoparticles. The strongest reflection from the (311) diffraction peak indicate of a cubic spinel structure. The reflection from other planes (022), (040), (151) and (044) also correspond with a cubic unit cell^38^.

**Table 2:** Peak list of Fe_3_O_4_ NPs. All the peaks of XRD patterns were analyzed and indexed using ICDD data base.

Pos (°2θ)	Height (cts)	FWHM left (°2θ)	d-spacing (Å)	Rel. Int (%)	Tip width	Matched by
30.2360	123.24	0.5117	2.95596	47.15	0.6140	Fe_24_O_32_
35.5989	261.39	0.3582	2.52199	100.00	0.4298	Fe_24_O_32_
43.2865	68.51	0.4093	2.09024	26.21	0.4912	Fe_24_O_32_
57.2379	63.41	0.7164	1.60953	24.26	0.8596	Fe_24_O_32_
62.8740	79.50	0.5117	1.47813	30.41	0.6140	Fe_24_O_32_

It can be noticed from XRD pattern and Table 2 that the 2θ position, d-spacing and intensity of the diffraction peaks are in good agreement with the standard pattern for Ref. Code 96-900-5841.

### Testing of bacterial sensitivity to NPs

To determine the sensitivity of *E. coli* strains to used NPs, bacteria were grown in the presence of Fe_3_O_4_ NPs (100 μg mL^−1^) and Ag NPs (10 μg mL^−1^), after which various dilutions (10^6^–10^9^ folds) of bacterial suspension were applied^20^. Then 100 μL of each sample was transferred on nutrient agar plates, which were incubated at 37 °C. Nutrient agar plates contained peptone media with 1.5% bacteriological agar. The numbers of bacterial viable colonies were counted after 24 h of incubation for determination of CFUs presented in each sample by: *T* = 10 × *n* × 10^*m*^, where *n* is the number of bacterial viable colonies, *m* is the number of dilution. NPs free plates incubated under the same conditions were used as control^20^.

### Determination of H^+^-fluxes through bacterial membrane

The H^+^-flux through the bacterial membrane in whole bacterial cells was determined using appropriate selective electrode (HJ1131B, HANNA Instruments, Portugal), as described^11,20,21^. Bacteria were cultivated in the presence of Fe_3_O_4_ NPs (100 μg mL^−1^) or Ag NPs (10 μg mL^−1^). Bacterial cells were transferred into the 150 mM Tris–phosphate buffer (pH 7.5), and then energy source—glucose (11 mM) was added. The H^+^-flux was expressed as a change in the ion external activity in mmol H^+^ per min per 10^10^ cells^11,20^. The bacterial cultures were incubated with 0.2 mM* N*,*N′*-dicyclohexylcarbodiimide (DCCD), an inhibitor of the F_O_F_1_-ATPase, for 10 min^11^.

### ATPase activity assay

Bacterial membrane vesicles were obtained by the Kaback method, as described^21,32^. ATPase activity was determined by amount of inorganic phosphate (P_i_), liberated after adding 3 mM ATP to membrane vesicles^11,21,32^. P_i_ was measured by the Tausski and Shorr method, as described^11,32^. ATPase activity was expressed in nmol P_i_ per µg protein per min. Bacteria were cultivated in the presence of Fe_3_O_4_ NPs (100 μg mL^−1^) orAg NPs (10 μg mL^−1^). For DCCD studies, membrane vesicles were incubated with 0.2 mM DCCD for 10 min.

### The medium pH, redox potential and H_2_ yield determinations

The pH of the medium was measured during bacterial growth at certain time intervals (from 0 to 6 h) by a pH-meter (HANNA Instruments, Portugal) with pH-selective electrode (HJ1131B), as described^20,33^. The initial pH was adjusted at 7.5 ± 0.1 by 0.1 M NaOH or 0.1 M HCl. The medium redox potential (*E*_*h*_) was measured during bacterial growth using a pair of redox [platinum (Pt) and titanium–silicate (Ti–Si)] electrodes, as described^20,33^. *E*_*h*_ kinetics determined using the pair of redox electrodes during culture growth gives information about main redox processes and also H_2_ evaluation^20,33^. The H_2_ yield in *E. coli,* cultivated in the presence of Fe_3_O_4_ NPs (100 μg mL^−1^) or Ag NPs (10 μg mL^−1^), was calculated by the decrease of *E*_*h*_ to low negative values during bacterial growth, as described early^21,33^, and expressed in mmol H_2_ per L.

### Reagents, data processing and others

Yeast extract, peptone, Tris (aminomethane), Agar–Agar Kobe from Carl Roth GmbH (Germany); glucose, DCCD from Sigma Aldrich (USA), and other reagents of analytical grade were used. The average data are presented from 3 independent experiments; error bars are presented on figures. The validity of the differences between different series of experiments was evaluated by Student criteria (*P*)^20^.
