# Author Correction: Comparable antibacterial effects and action mechanisms of silver and iron oxide nanoparticles on *Escherichia coli* and *Salmonella typhimurium*

**DOI:** 10.1038/s41598-021-90986-x

**Published:** 2021-06-01

**Authors:** Lilit Gabrielyan, Hamlet Badalyan, Vladimir Gevorgyan, Armen Trchounian

**Affiliations:** 1 Department of Medical Biochemistry and Biotechnology, Russian-Armenian University, 0051 Yerevan, Armenia; 2 Department of Biochemistry, Microbiology and Biotechnology, Biology Faculty, Yerevan State University, 0025 Yerevan, Armenia; 3 Department of General Physics and Astrophysics, Yerevan State University, 0025 Yerevan, Armenia; 4 Department of Technology for Materials and Electronic Technique Structures, Russian-Armenian University, 0051 Yerevan, Armenia

Correction to: *Scientific Reports* 10.1038/s41598-020-70211-x, published online 04 August 2020

The original version of this Article contained errors in Figures 5A and 6A, where the incorrect TEM images of nanoparticles were used.

The original Figures 5 and 6 and accompanying legends appear below.

The original Article has been corrected.

**Figure 5:**
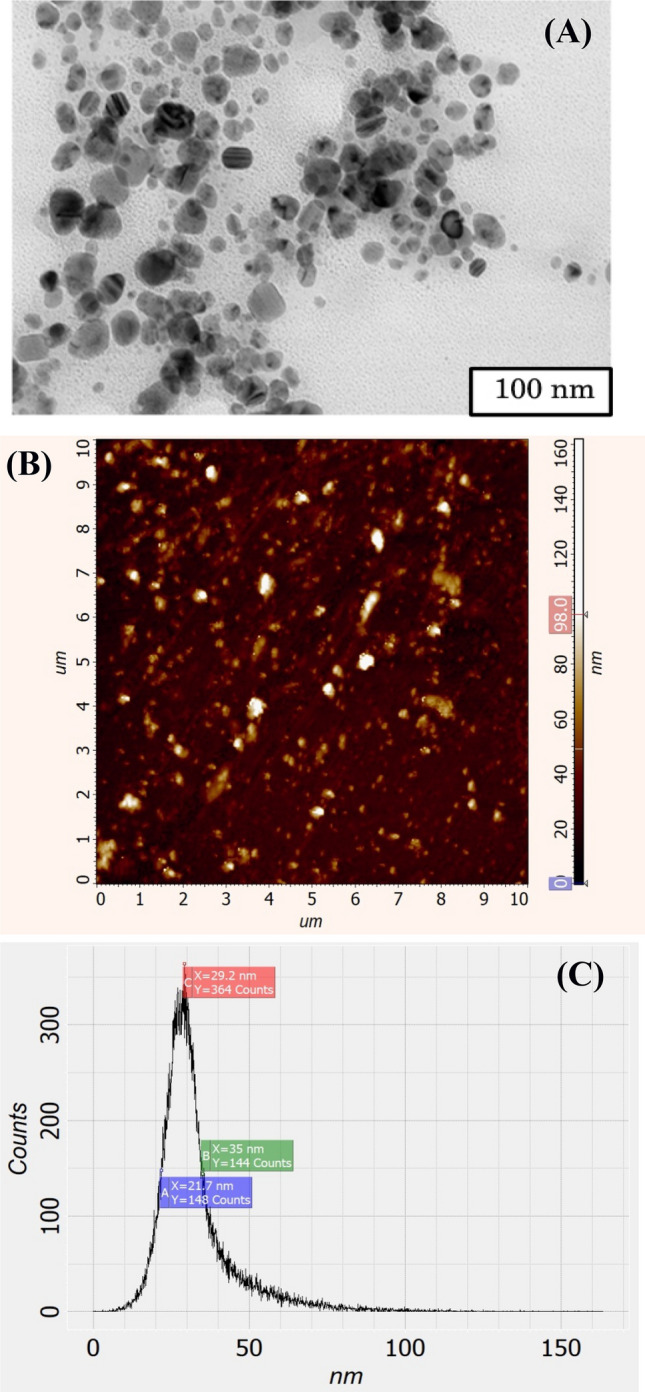
TEM image (**A**) and atomic force microscopy of Ag NPs using appropriate microscope (**B**). Ag NPs distribution in the colloid solution depending of their size (**C**). For details, see “Materials and methods”.

**Figure 6:**
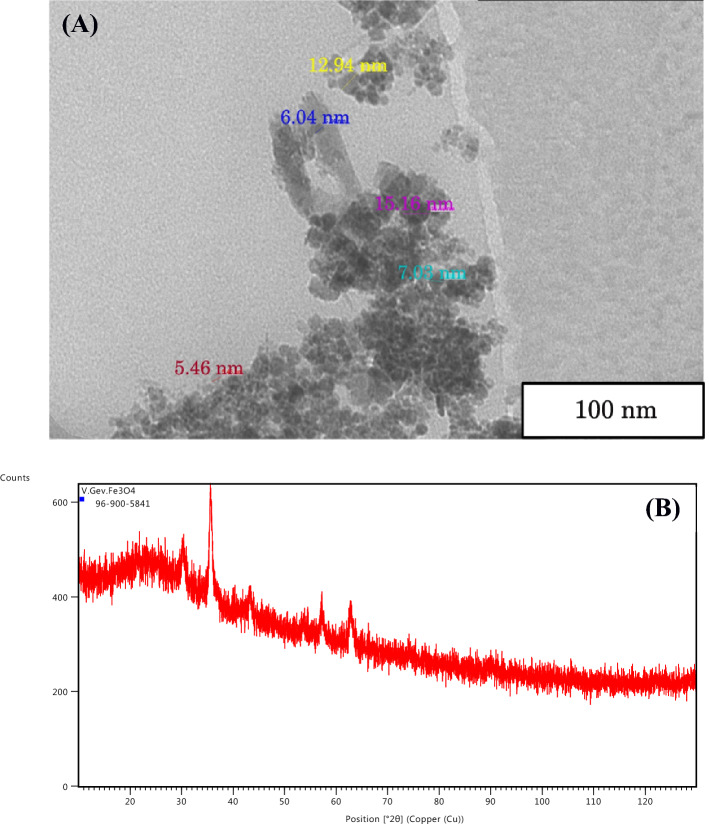
TEM image of synthesized Fe_3_O_4_ NP (**A**). The sizes of some nanoparticles indicated immediately in the figure. XRD pattern of the Fe_3_O_4_ NPs (**B**).

